# Robust moving-blocker scatter correction for cone-beam computed tomography using multiple-view information

**DOI:** 10.1371/journal.pone.0189620

**Published:** 2017-12-21

**Authors:** Cong Zhao, Xi Chen, Luo Ouyang, Jing Wang, Mingwu Jin

**Affiliations:** 1 Dept. of Physics, University of Texas at Arlington, Arlington, TX, United States of America; 2 Dept. of Radiation Oncology, University of Texas Southwestern Medical Center, Dallas, TX, United States of America; North Shore Long Island Jewish Health System, UNITED STATES

## Abstract

Scatter contamination is one of the main sources of decreasing the image quality in cone-beam computed tomography (CBCT). The moving blocker method is economic and effective for scatter correction (SC), which can simultaneously estimate scatter and reconstruct the complete volume within the field of view (FOV) from a single CBCT scan. However, at the regions with large intensity transition in the projection images along the axial blocker moving direction, the estimation of scatter signal from blocked regions in a single projection view can produce large error and cause significant artifacts in reconstructed images and null the usability of these regions. Furthermore, blocker edge detection error can significantly deteriorate both primary signal and scatter signal estimation and lead to unacceptable reconstruction results. In this study, we propose to use the adjacent multi-view projection images to jointly estimate scatter signal more accurately. In return, the more accurately estimated scatter signal can be utilized to detect blocker edges more accurately for greatly improved robustness of moving-blocker based SC. The experimental results using a Catphan phantom and an anthropomorphic pelvis phantom CBCT data show that the new method can effectively suppress the estimation errors of scatter signal in the fast signal transition regions and is able to correct the blocker detection errors. This development will expand the utility of moving-blocker based SC for the target with sharp intensity changes in the projection images and provide the needed robustness for its clinical translation.

## 1. Introduction

Come-beam computed tomography (CBCT) is being widely used as an image guidance tool in radiation therapy nowadays. The quality of CBCT image is important to localize and delineate the tumor and to define relevant volumes when the patient is in the treatment position [1]. However, due to the use of the flat panel detector (FPD), the large amount of scatter signal deteriorates the projection images and greatly reduces the quality of CBCT images [2]. The manifest shading artifacts can lead to decreased image contrast and inaccurate CT number, which makes it difficult to contour the target and other volumes and to calculate the correct dose [3]. Therefore, scatter correction (SC) is critically important to overcome these problems of CBCT for a precise adaptive radiation therapy.

Different strategies have been investigated to correct the scatter contamination in CBCT projection data, e.g. suppression of scatter signal during the acquisition using anti-scatter grids [4–7] and correction of scatter signal in projections using computational methods, such as analytical models or Monte Carlo simulations [8–13]. There have been growing interests in developing measurement-based scatter correction, such as blocker-based methods [14–26]. These methods assume that the scatter is a low frequency signal and derive the scatter signal from the attenuator-blocked regions within the projection images, thus avoiding the computationally intensive Monte Carlo simulation [4, 9, 10, 27] or the oversimplification of analytical methods [11]. The fixed-blocker methods suffer the problems of the requirement of additional scans or the reduced imaging volume. The moving blocker method [20, 25] overcomes these limitations of fixed-blocker methods and is cable of simultaneously estimating the scatter signal and reconstructing the entire volume within the field of view (FOV) from a single CBCT scan. As an extra benefit, the radiation dose can be greatly reduced, e.g. by half if the same blocker width and pitch were used. The moving blocker method was further advanced recently by a deconvolution method with an aim to improve the scatter signal estimation in the blocked regions by reducing the primary signal contamination [28]. Nevertheless, there are still a couple of issues with the moving blocker method, namely the large estimation errors in the regions with large scatter signal transition and/or insufficient scatter samples in the projection images (called “boundary effect” as follows) and the vulnerability to blocker detection errors (called “robustness” as follows). These errors can cause significant artifacts in corresponding regions in reconstructed images and possibly make them useless.

In our previous study [25], we constructed a moving blocker system to show the effectiveness of simultaneous SC and volumetric reconstruction [20]. The original moving-blocker SC method uses scatter measures in the blocked regions to estimate the scatter signal for unblocked regions for each individual projection view (which is called “single-view” SC method in the following context). Such a treatment has achieved much improved image quality and more accurate CT numbers compared to CBCT without SC in most cases. However, there are a couple of issues arose from the single-view SC method: 1) boundary effect: image slices near the boundary of the object that are deteriorated by severe artifacts because the scatter signal is changing fast in these regions which violate the low-frequency assumption of scatter signal or an extrapolation is needed when the blocked regions (scatter samples) do not cover one end of the axial FOV; and 2) robustness: the SC performance is prone to the blocked and unblocked region detection errors and thus the accurate blocker position is required for the success of the moving-blocker SC method. However, for CBCT projection data, especially for half-fan scan for a big-size object, it could be challenging to find the blocker edge because the projection image has very limited contrast at the phantom regions. The wrong edge detection can lead to unacceptable reconstruction results with severe artifacts. To address these issues and make the moving-blocker SC method for a wider and more robust application, in this study, we propose a multi-view SC method, which combines the adjacent projection views to jointly estimate the scatter signal for the current projection view. As long as the projections are densely acquired around the object (e.g. 670 projections for a full 360° rotation used in this study), the scatter signal in adjacent projection views does not change much, because the angular difference among adjacent projection views is very small and the scatter signal contains little high-frequency information. We hypothesize that these adjacent projection views can provide complementary scatter signal in different regions along the rotation axis when the blocker is moving with the gantry rotation. Thus, a more accurate estimate of the scatter signal in large intensity transition regions can be achieved as the sampling rate is significantly increased (3~5 times depending on the number of adjacent views used). In order to overcome the adverse effect of the blocker detection errors, the estimated scatter from adjacent projection views can also be used for more accurate and robust edge detection in low contrast regions.

Both the Catphan phantom and an anthropomorphic pelvis phantom were used to test our multi-view SC method. We compared the quality of reconstructed images and CT numbers for different SC methods (no SC, single-view SC, and multi-view SC) using multi-detector CT (MDCT) images and CT numbers as a benchmark. This study aims to make the moving blocker-based SC method more robust and extend its effectiveness for the target near the boundary of the field of view or with sharp intensity changes in the projection images.

## 2. Methods and experiments

### 2.1. Moving-blocker based SC method

**Fig 1:**
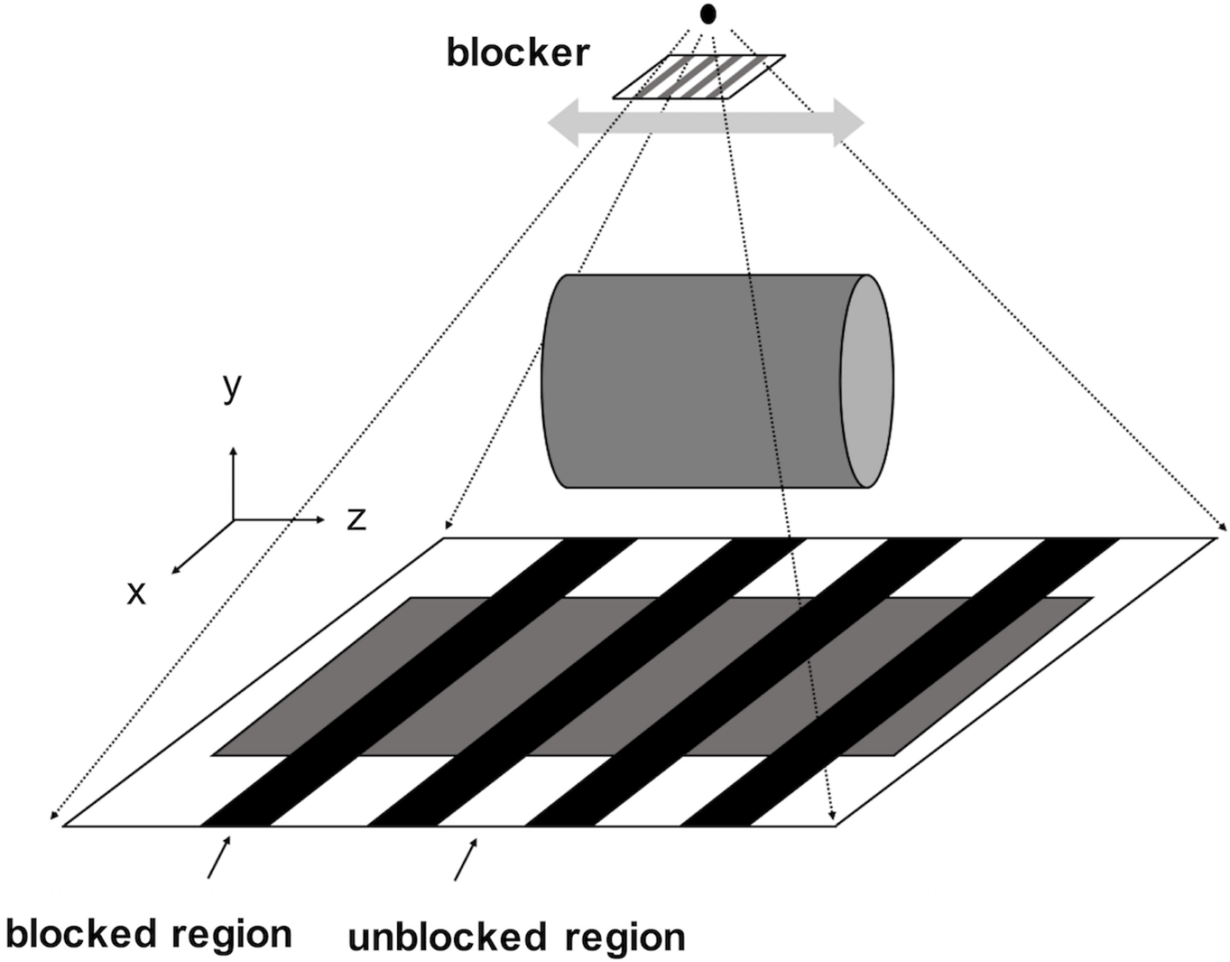
The moving blocker CBCT system. The rotational axis of the gantry is z and the blocker is moving back-and-forth in z direction. The blocked region detects the scatter signal and the unblocked region detects the total signal, i.e. the sum of the primary and scatter signals.

**Fig 2:**
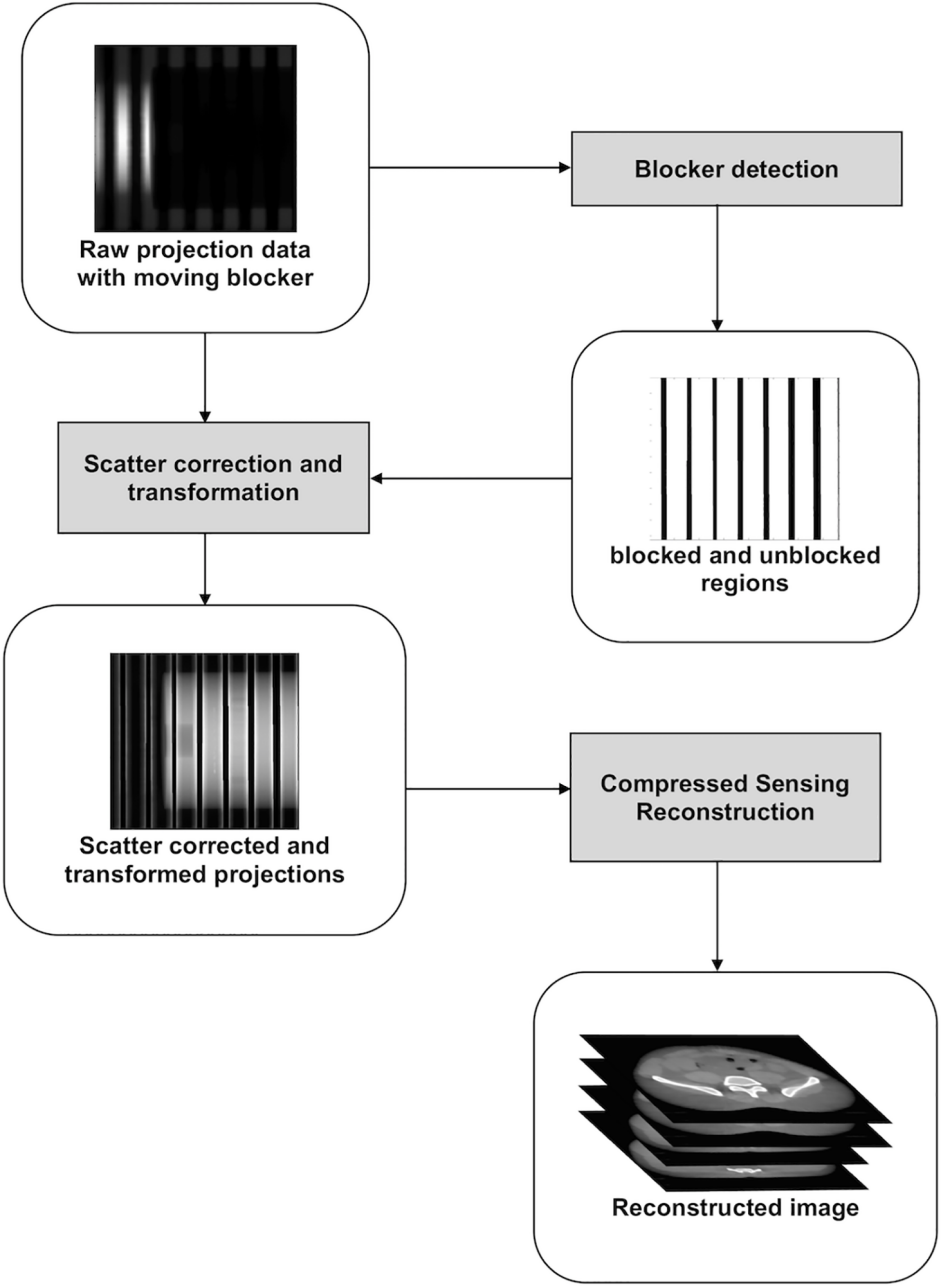
The process of the moving blocker CBCT reconstruction with scatter correction.

The moving-blocker based SC method has been proposed to simultaneously estimate scatter signal and reconstruct the complete volume within the FOV from a single CBCT scan [20, 25]. A moving blocker system consists of several lead strips inserted between the X-ray source and the object. These lead strips are aligned perpendicularly to the gantry rotation axis and move back and forth along it while the source and detector rotating as shown in Fig 1. The method assumes that the blocked regions only receive the scatter signal, while the unblocked regions contain the sum of the primary and scatter signal (“total signal”). A flow chart of the moving blocker scatter correction process is shown in Fig 2. As the scatter signal is generally a smooth low-frequency signal, the scatter signal in the unblocked regions can be estimated by interpolating the scatter signal in the blocked regions. Then the primary signal in the unblocked regions can be recovered by subtracting the estimated scatter signal from the total signal. As the blocker is moving along the gantry rotation axis, the missing data (in blocked regions) are different in different projection views, which is equivalent to a fewer view projection data acquisition, and the reconstruction can be effectively solved by the compressed sensing techniques. Accordingly, a total variation (TV) constrained iterative method can be used to reconstruct scatter corrected images for the full volume in the FOV [20, 29–34]. Specifically, the discrete CBCT image of the linear attenuation coefficient *μ* is solved by minimizing the TV function *f*(*u*):

μ=argmin(f(μ)), (1)

$$f(\mu )=\sum_{x,y,z}\sqrt{{\left(\mu_{x,y,z}-\mu_{x-1,y,z}\right)}^{2}+{\left(\mu_{x,y,z}-\mu_{x,y-1,z}\right)}^{2}+{\left(\mu_{x,y,z}-\mu_{x,y,z-1}\right)}^{2}},$$

where *x*, *y*, and *z* are the three-dimensional coordinates of each voxel of the reconstructed image. Eq (1) is enforced by the standard steepest descent algorithm and subjected to the data fidelity and the non-negativity constraints:

$$|A\mu -\tilde{p}|\leq \varepsilon ,$$ (2)

μ≥0, (3)

where $\tilde{p}$ is the log-transformed projection after scatter correction, *A* is the forward projection system matrix and *ε* is an error bound determined by the data noise and imaging model error. The constraint in Eq (2) is enforced by the standard algebraic reconstruction technique (ART):

$$\mu_{i}^{\left(k+1\right)}=\mu_{i}^{\left(k\right)}+\lambda a_{ij}\left[\frac{p_{j}-\sum_{i}a_{ij}\mu_{i}^{\left(k\right)}}{\sum_{i}a_{ij}^{2}}\right],$$ (4)

where *a*_*ij*_ is the intersection length of projection ray *j* with pixel *i*, calculated by fast ray-tracing technique [35], *k* is the iterative step and *λ* is the relaxation factor. After each ART update of Eq (4), the resulting image was updated according to Eq (1). More details of reconstruction can be found in Ref. [20, 25].

### 2.2. Issues with single-view moving-blocker SC method (SVSC)

#### 2.2.1. Boundary effect

**Fig 3:**
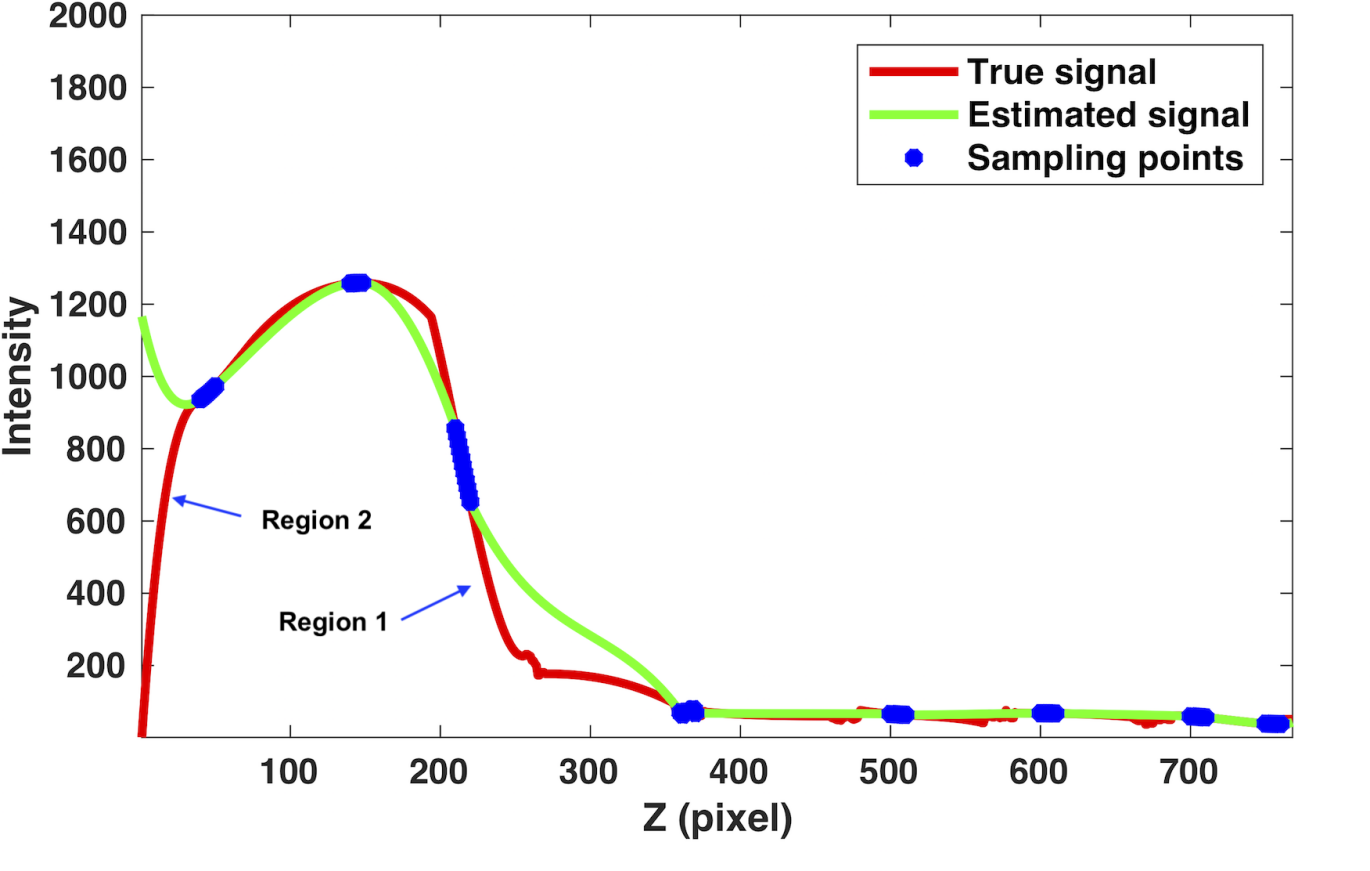
The boundary effect due to the interpolation and extrapolation using limited sampling points in the single view SC (SVSC) method for the Catphan phantom.

If the low-frequency smooth condition of scatter signal is violated, such as at the boundaries between the object and the air or between bone and soft tissues inside body where the scatter signal changes fast, the current scatter estimation method using a single projection view may cause scatter estimation errors in the unblocked regions. In the projection data of these regions, some views may contain accurate scatter information if they are blocked, while some views are lack of direct measurements as they are unblocked. For these (unblocked) views, the interpolation has to be applied to estimate the scatter signal. However, the limited sampling points in a single projection view are not sufficient to recover the transition of the scatter signal. As shown in Fig 3 for a line profile of projection data of CatPhan (red line: true scatter signal; blue dots: sampling points from the blocked region; green line: estimated signal by cubic B-spline interpolation of blue dots), there are two kinds of problems due to the boundary effect. In region 1, the insufficient sampling points cause the interpolation to overestimate the scatter signal. In region 2, the extrapolation has to be used due to the lack of sampling points at the end and can introduce large estimation errors. Consequently, these significant scatter estimation errors will lead to inaccurate SC and severe artifacts in the reconstructed images.

#### 2.2.2. Robustness

**Fig 4:**
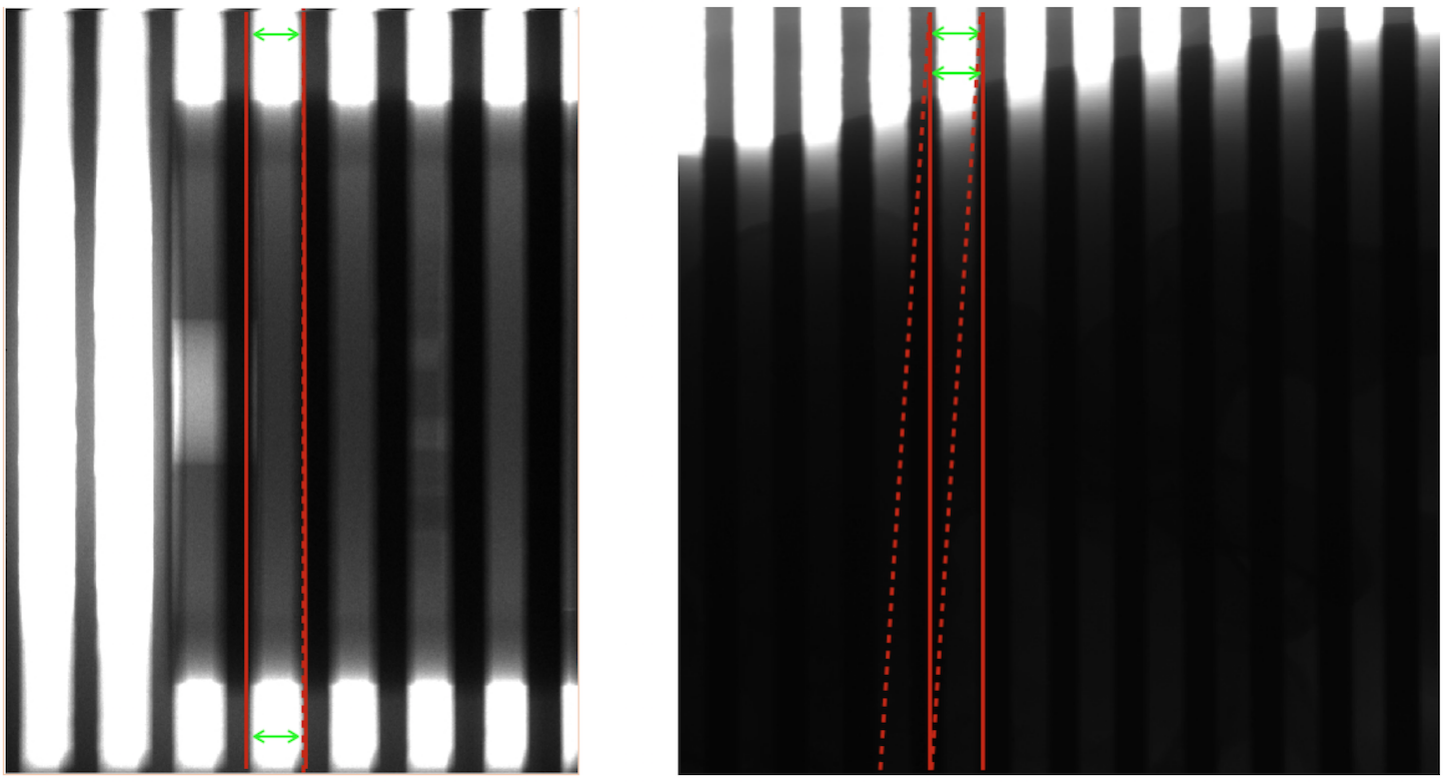
Blocker edge detection for a small object (left) and a large object (right). The green arrows denote the position for reliable edge point detection in high-contrast regions. The edge is estimated as the line connecting two edge points. The red solid lines represent the true blocker edges and the red dashed lines are the estimated edges.

In the SVSC method, the blocker edges have to be determined for each view so that the blocked regions and unblocked regions can be separated for scatter estimation and reconstruction, respectively. Therefore, the success of the moving-blocker SC method highly depends on the accuracy of the detection of the blocker edges. Ideally, the edges of blockers can be detected in each line profile along the z direction by intensity thresholding. However, this can cause erroneous detection in areas with low contrast. In practice, by assuming a straight line of the blocker edge, edge detection can be performed at two z lines to get the straight-line edge, so called “two-point method”. This two-point method is easily achievable for a high contrast projection image (Fig 4 left), where two edge points far away from each other along one blocker edge. In Fig 4 left, the full-fan mode was used, suitable for the small size of the phantom (Catphan). Two points, one on the top and the other at the bottom, can be used to calculate the blocker edges and result in very accurate edge detection. In this case, both the true edges (solid red lines) and estimated edges (dashed red lines) overlapped with each other. However, when imaging a large-size object, the blocker edge detection can be erroneous due the large area of low image contrast (Fig 4 right). In Fig 4 right, due to the large size of the phantom (Pelvis phantom), the half-fan mode was used, and consequently only few rows on the top of the projection image provide sufficient contrast for reliable blocker edge detection as the bottom part’s contrast is too low to identify the blocker edge reliably. Since the two edge points are too close, a little error can propagate along the rest part of the straight line and generate large blocker edge estimation errors (Fig 4 right, red dashed lines) from the true edges (Fig 4, red solid lines). This erroneous blocker edge detection caused by the two-point method can lead to artifacts in reconstructed images since both scatter signal and primary signal are estimated incorrectly.

### 2.3. Multi-view moving blocker SC method (MVSC)

The multi-view SC (MVSC) method is proposed to address the issues with the single-view SC (SVSC) method using the moving blocker. The MVSC is to use adjacent projection views to jointly estimate the scatter for the current projection view. Should the angles be small among the neighboring projection views used for this joint scatter estimation, the change of the scatter profile can be negligible. The location change of the blocked regions in these projection views due to the axial blocker movement can be exploited to boost the sampling rate of the scatter signal, which is only sparsely sampled in the SVSC method due to the limited lead strip width and pitch of the blocker. As more samples are used for the interpolation of the scatter signal, more accurate scatter estimation can be achieved by MVSC than that by SVSC. This is particularly beneficial for the regions with large scatter signal transition. In addition, the complimentary scatter information from the neighboring views can be used to cross check for the wrong blocker detection and mitigate the scatter estimation errors. On the other hand, the reconstruction process is kept intact in MVSC so that more accurate and robust reconstruction can be achieved through more accurate and robust scatter correction. The estimated scatter signal for *i*^th^ projection view through interpolation can be expressed as:

$$S_{i}^{SE}(z)=\sum_{k=1}^{K}a_{k}W_{k}(z),$$ (5)

where *W*_*k*_(*z*) and *a*_*k*_ are the basis functions and corresponding weights and the lateral dimension is omitted for clarity. The smoothness of estimated signal is enforced by the choice of the basis functions. In this work, the cubic B-spline functions were used. For the proposed MVSC method, the optimal weights can be found by minimizing the following least-squares objective function:

$$a^{*}=\arg\underset{a}{\min}\sum_{j\in ℵ(i)}\sum_{n=1}^{N}{\left\|S_{j}^{SS}(z_{jn})-\sum_{k=1}^{K}a_{k}W_{k}(z_{jn})\right\|}^{2},$$ (6)

where ℵ(*i*) is the neighboring projection views for the *i*^th^ view (including *i*), $S_{j}^{SS}(z_{jn})$ is the observed scatter signal in the *n*^th^ blocked region of the *j*^th^ projection view, and **a** is the vector of all weights *a*_*k*_. In this work, the third order polynomials were used for the basis functions *W*_*k*_(*z*) and the weights *a*_*k*_ were estimated using Newton’s method. When ℵ(*i*) = {*i*}, the MVSC method is collapsed down to SVSC as only one projection image is used for the scatter estimation. In contrast, the MVSC method uses more than one projection image, e.g. ℵ(*i*) = {*i* -1, *i*, *i* +1} if three adjacent views are used. The caveat is that these adjacent project views shall span only a small angle to avoid any significant scatter signal changes among these views. It is worth noting that the proposed multi-view scatter correction (MVSC) method still uses projections from a single rotation acquisition, thus the imaging dose and the acquisition time are the same as the single-view scatter correction (SVSC) method. Essentially, the MVSC method does not change the CBCT data acquisition of the SVSC method, using the same total number of projection views for reconstruction and scatter correction. The key difference of two methods is that the SVSC method uses only one projection view for scatter estimation, while the MVSC method uses several adjacent projection views for scatter estimation.

**Fig 5:**
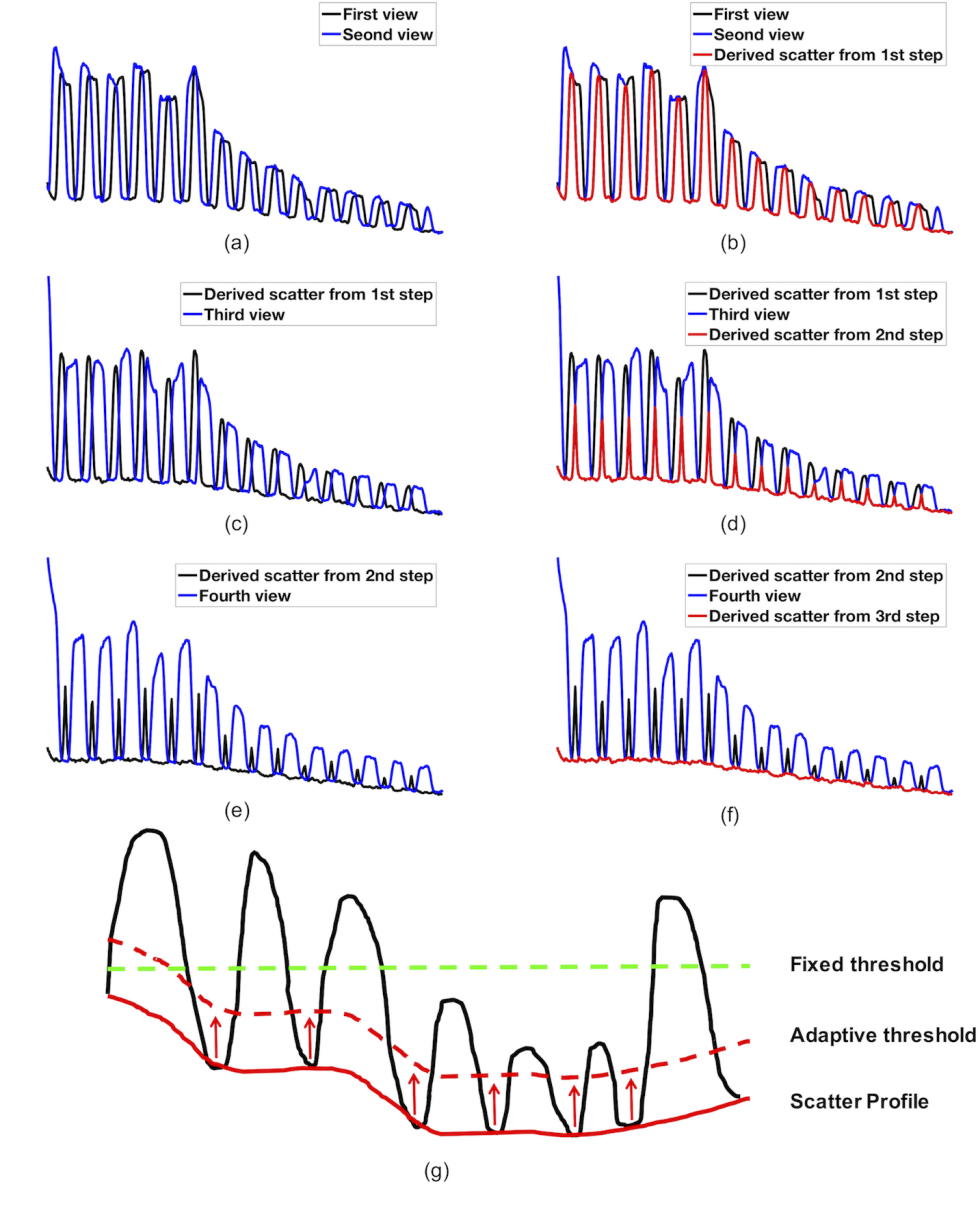
Multi-view scatter estimation and adaptive threshold for blocker edge detection. (a)-(f): the procedure to get the scatter profile (vertical axis) using the lowest signal in the signal profiles along the axial direction (horizontal axis) from multiple adjacent views (4 in this case); and (g) adaptive threshold (dashed red line) is derived from the scatter profile (red line) for more accurate and robust blocker edge detection, while the fixed threshold (green dashed line) fails to detect some edges in this case.

In addition, the scatter signal can be estimated from multiple projection views directly so that the wrong blocker edge detection (Fig 4) by the two-point method can be avoided. By composing the signal profiles from several neighboring views, the scatter profile can be readily estimated without the need of the blocker edge detection at first. The procedure is shown in Fig 5A and 5F with the signal profiles (vertical axis) along the axial direction (horizontal axis): 1) compare signal profiles from two adjacent views (blue and black lines in Fig 5A) and keep the lower signal in each position to get the derived signal profile (red line in Fig 5B); 2) compare the derived signal profile from the first step (black line in Fig 5C, same as red line in Fig 5B) with that from the third adjacent view (blue line in Fig 5C) and keep the lower signal in each position to get the derived signal profile (red line in Fig 5D); 3) repeat step 2) by comparing a couple of more adjacent views (Fig 5E) to get the final scatter profile (red line in Fig 5F) without detecting the blocker edges in the first place. Once the scatter signal (red solid line in Fig 5G) was estimated, an adaptive threshold (red dash line in Fig 5G) can be used to detect the blocker edges for the determination of the unblocked region, and then subsequent scatter correction and reconstruction. The fixed threshold (green dashed line in Fig 5G) derived from the mean of the intensity fails to detect all blocker edges in the cases, where the intensity profile has a large fluctuation. Note that the valley point detection using a single signal profile for scatter estimation is not reliable due to the large signal fluctuation and noise.

### 2.4. Experimental data and processing

To evaluate the MVSC method, we acquired the CBCT projection data of a commercial calibration phantom CatPhan® 600 (ThePhantom Laboratory, Inc., Salem, NY) using a Varian TrueBeam OBI system (Varian Medical Systems, Inc., Palo Alto, CA) (with a manual shift to mimic the moving blocker acquisition) and an anthropomorphic pelvis phantom CIRS 801-P (Computerized Imaging Reference Systems Inc., Norfolk, VA) using an Elekta Synergy XVI system (Elekta AB, Stockholm, Sweden) both with and without the moving blocker. The source-to-axis distance is 1000 mm and the source-to-detector distance is 1500 mm with a full 360° coverage in approximately 1 minute for the Varian TrueBeam OBI system. The source-to-axis distance is 1000 mm and the source-to-detector distance is 1536 mm with a full 360° coverage in approximately 2 minutes for the Elekta Synergy XVI system. There are 660~680 projection views acquired and each projection contained 512 × 512 pixels with a pixel size of 0.8 × 0.8 mm. We use full-fan scan for Catphan phantom (120kVp and 20 mA/10 ms) and half-fan scan for the pelvis phantom (120kVp and 80 mA/20 ms). The multi-detector CT (MDCT) (Philips Healthcare, Nevada, US) images of both phantoms were also acquired to serve as the evaluation benchmark.

The blocker is inserted between the x-ray source and the phantom and moves back and forth during CBCT gantry rotation. The moving blocker consists of equal-width lead strips embedded on a 3 mm thickness acrylic board (120 × 180 mm) are aligned along the detector face. The lead strips are 3.2 mm in thickness, 3.2 mm in width and are placed with a 3.2 mm pitch for the Catphan phantom and a 9.6 mm pitch (for boundary effect) and the 3.2mm pitch (for robustness) for the pelvis phantom. To investigate the performance of the MVSC method on the boundary effect, we used the both the Catphan data and the anthropomorphic pelvis phantom data. The pelvis phantom data was used to demonstrate the robustness of the MVSC method to the blocker detection errors (exist in most of the views) since it is more challenging to detect the blocker edge for the half-fan scan of a large object (e.g. the pelvis phantom), while the full-fan scan of a small object (e.g. Catphan) is hardly a problem as detailed in Section 2.2.2 and Fig 4.

### 2.5. Evaluation metrics

To evaluate the performance of the different SC methods, we compare various reconstructed image slices of CBCT with SVSC and MVSC with the corresponding MDCT images, served as a gold standard. We also calculate the CT numbers in Hounsfield Unit (HU) in several regions of interest (ROIs) for a quantitative evaluation of reconstruction accuracy using different SC methods for the pelvis phantom data. Both the mean and standard deviation (STD) of CT numbers in the ROIs are presented. The overall accuracy in each ROI is measured by the root mean square error (RMSE) following the equation:

$${RMSE}_{ROI}=\sqrt{\sum_{i=1}^{N}{\left({CT}_{CBCT}^{i}-{CT}_{MDCT}^{i}\right)}^{2}/N},$$ (7)

Where ${CT}_{CBCT}^{i}$ and ${CT}_{MDCT}^{i}$ denote the CT number of the *i*^th^ pixel in the selected ROI for CBCT and MDCT, respectively, and *N* denotes the total number of pixels in the selected ROI.

## 3. Results

### 3.1 Scatter estimation and correction for the Catphan reconstruction

**Fig 6:**
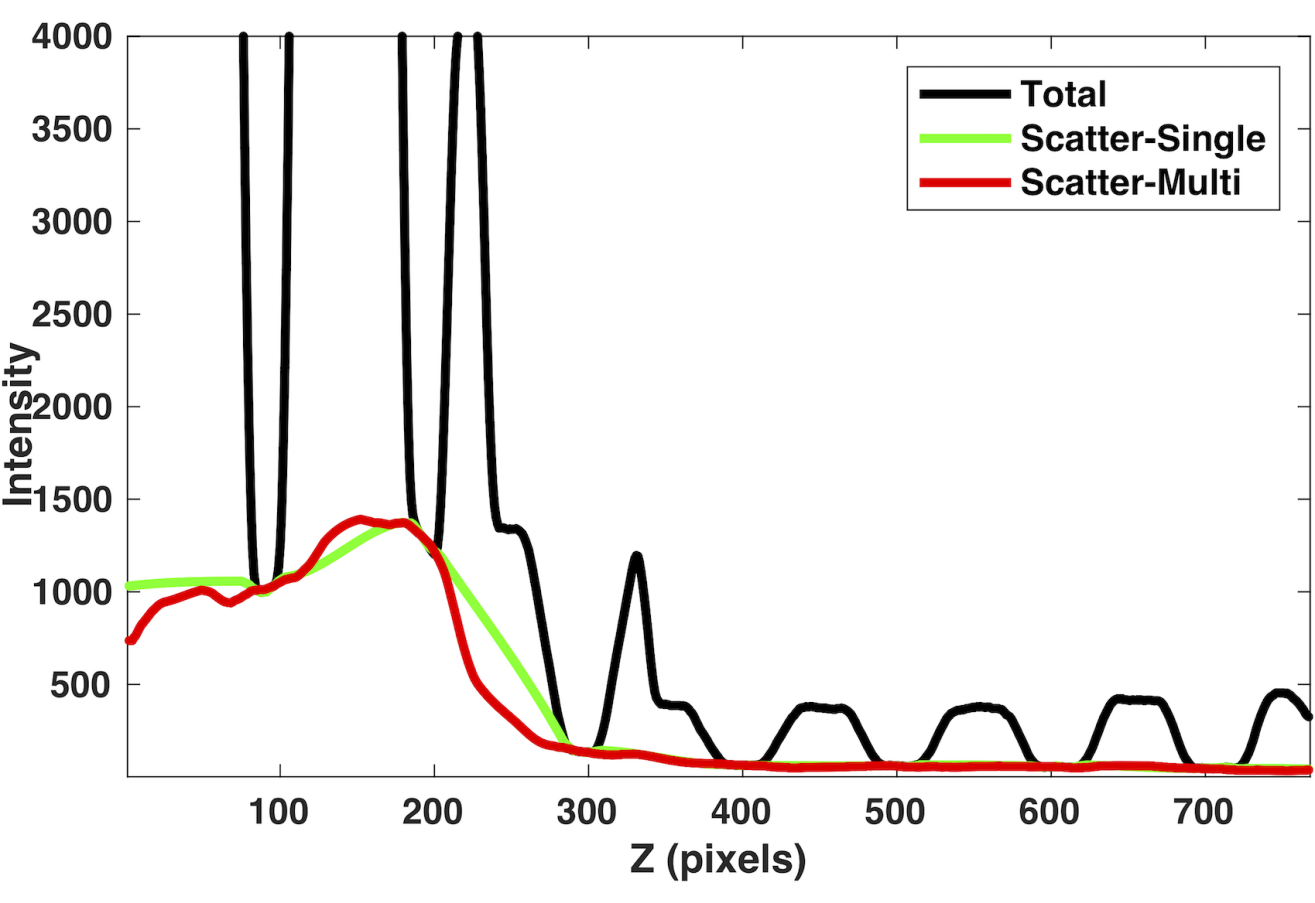
The comparison of scatter signal estimated from the single-view scatter correction (SVSC) method (green line) and the multi-view scatter correction (MVSC) method (red line) for the Catphan phantom (3.2 mm pitch blocker). The observed total raw signal is in black.

Shown in Fig 6 are the typical estimated scatter signal profiles using the SVSC method (green line) and the MVSC method (red line) using adjacent five views along with the observed total raw signal (black line) for the Catphan phantom. The SVSC method using only the current projection view for scatter estimation overestimated the scatter signal for the unblocked region between 200 and 300 along the horizontal axis (green line), thus leading to the underestimated primary signal. The MVSC method corrected this overestimation by utilizing more sample points from adjacent projection views and led to more gradual scatter transition (red line). It will be evident from the reconstructed images that this scatter estimation is more accurate.

**Fig 7:**
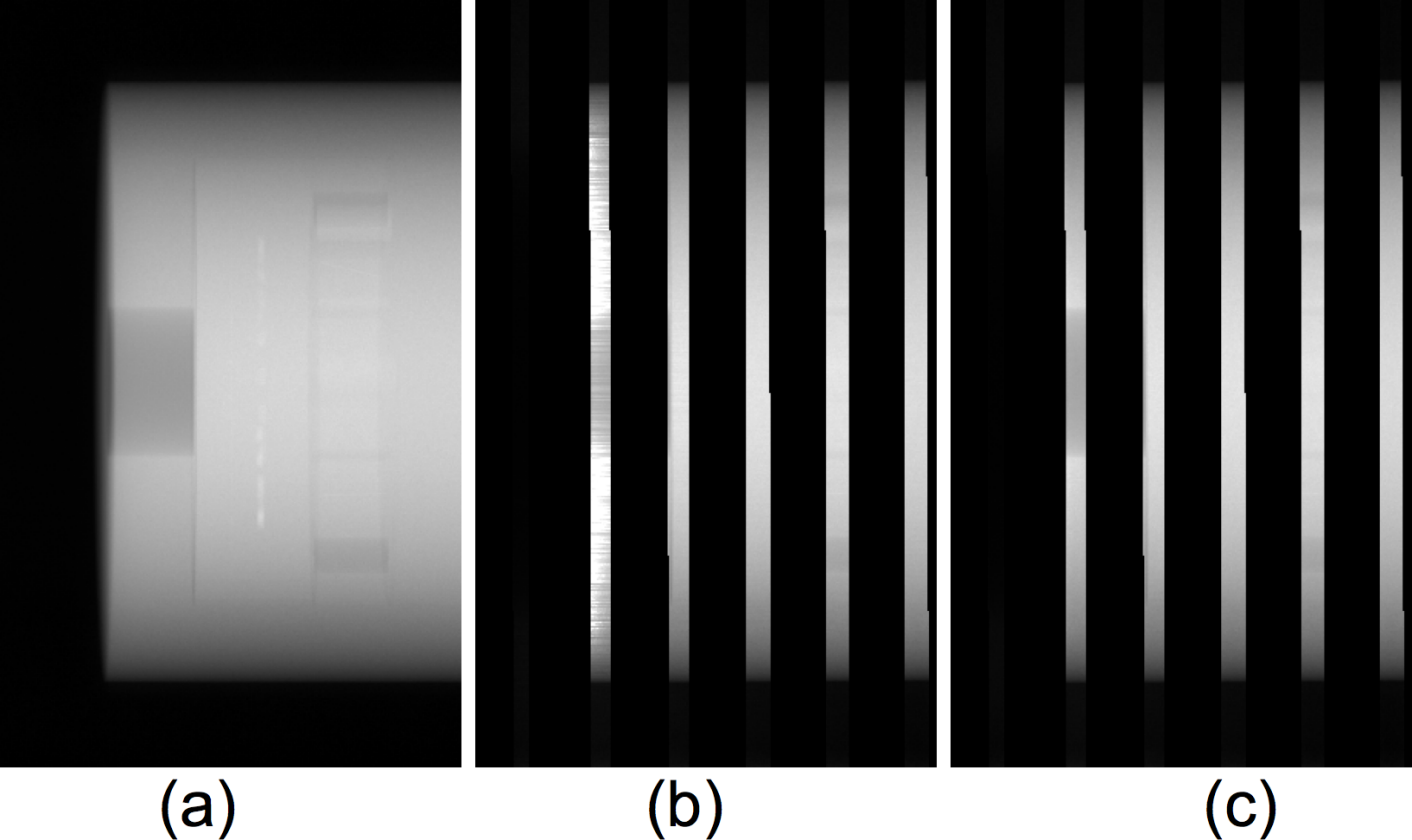
Projection images of the Catphan phantom (the line integral of attenuation coefficients by taking logarithm of inversely normalized X-ray fluence): a) without blocker; b) with SVSC; and c) with MVSC. Each image was normalized to its maximum intensity value, respectively.

In Fig 7, the projection images (the line integral of attenuation coefficients by taking logarithm of inversely normalized X-ray fluence) in a particular projection view are shown for: a) without moving blocker; b) SVSC; and c) MVSC. Severe bright strip artifacts can be observed in the unblocked region near the phantom boundary (the leftmost unblocked region, Fig 7B), where the overestimated scatter signal results in largely reduced primary signal, and thus much greater values of the line integral of attenuation coefficients. The MVSC method effectively eliminates these artifacts (the leftmost unblocked region, Fig 7C).

**Fig 8:**
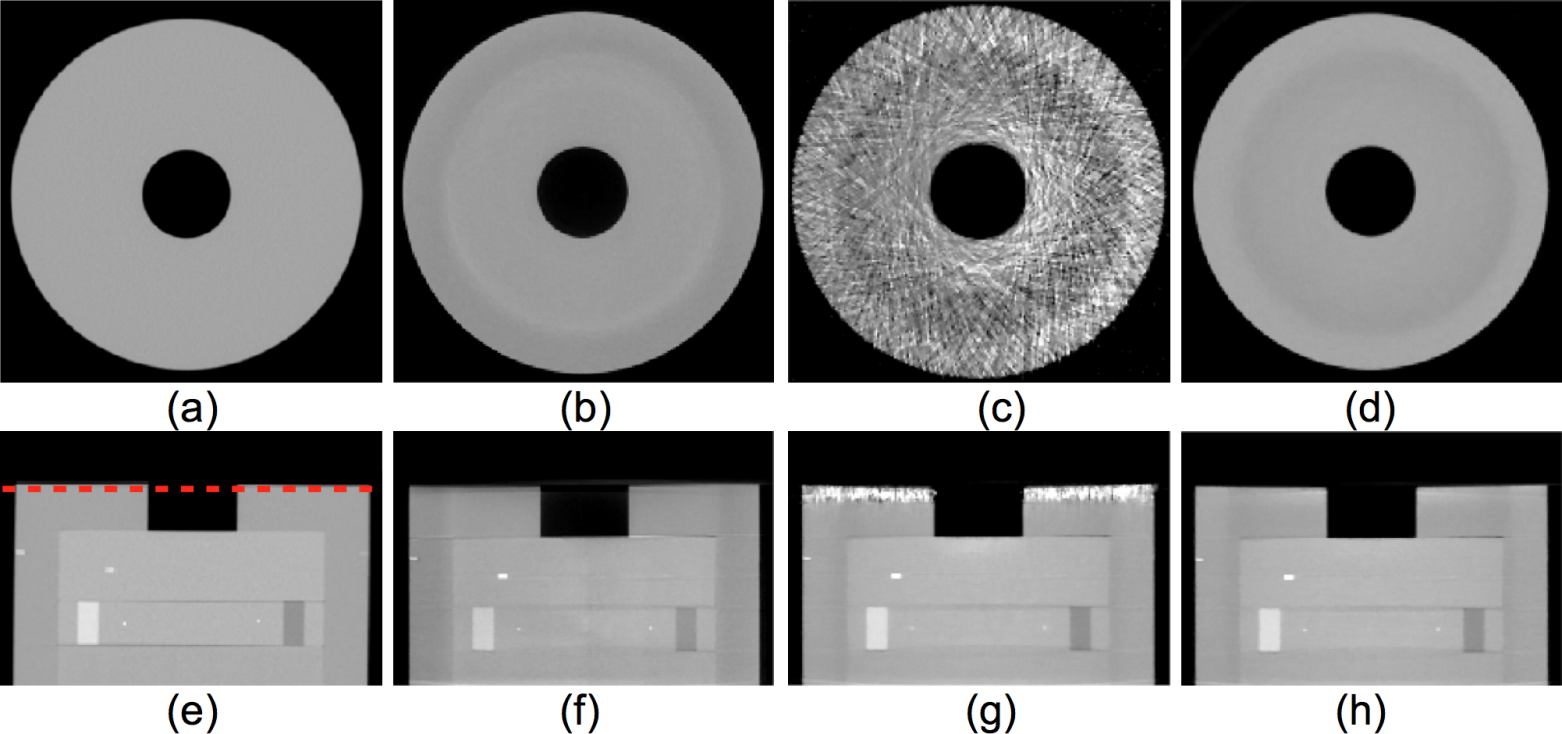
Reconstructed images for CBCT of the Catphan phantom (close to the axial left boundary in Fig 7): (a, e) benchmark fan-beam MDCT; (b, f) without SC; (c, g) SVSC; and (d, h) MVSC. The red dashed line in (e) labels the transverse slice location. The difference between without SC and MVSC can be better discerned through the line profiles in Fig 9. (Display window [−900, 500] HU).

**Fig 9:**
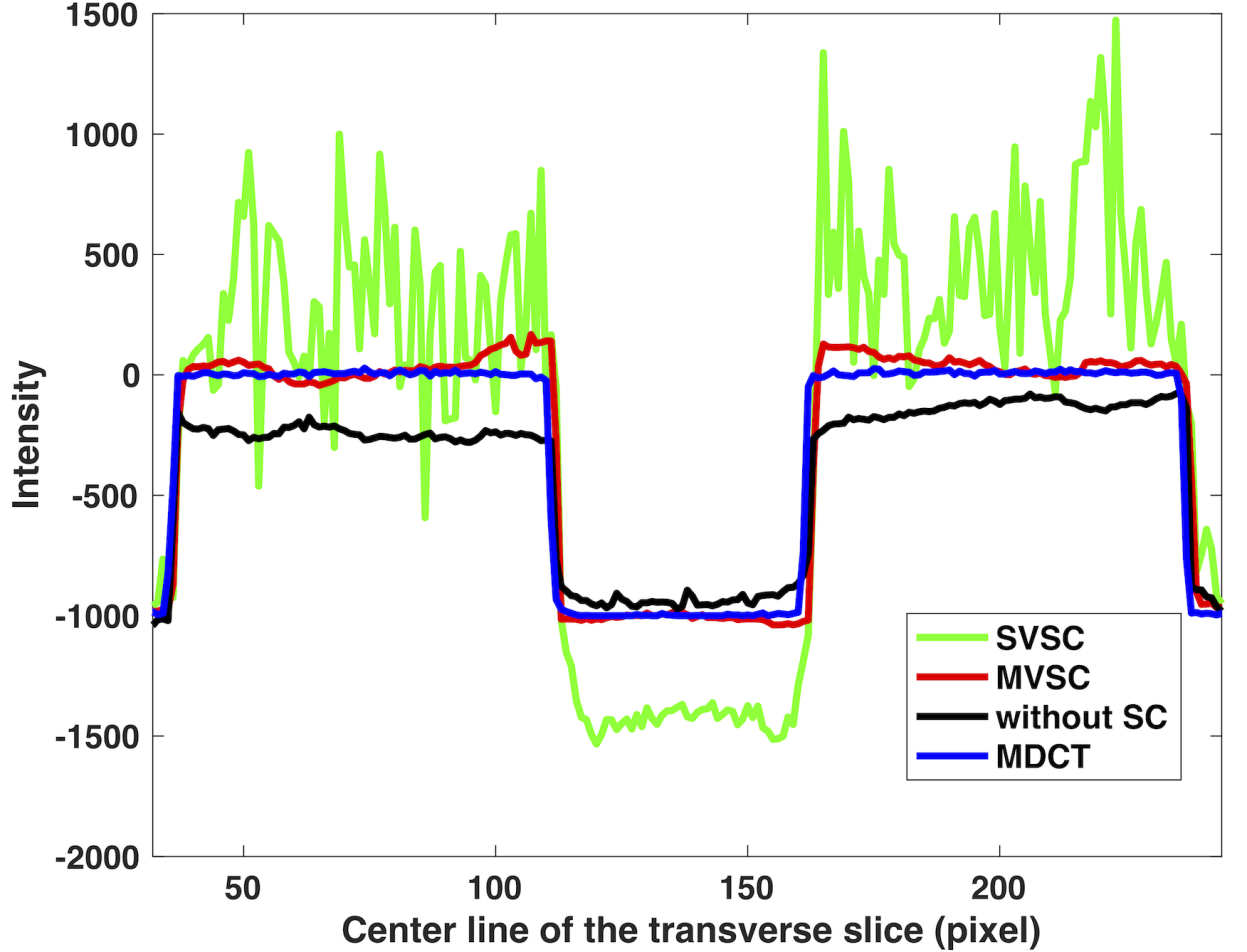
Line profile through the middle line of the transverse slice in Fig 8. The middle part is air and the CT number should be around -1000.

One transverse slice (first row) close to the phantom axial left boundary (Fig 8E, red dashed line) and one center coronal slice (second row) using different methods are shown in Fig 8. The reconstructed image without the moving blocker (Fig 8B and 8F) shows no significant artifacts, but suffers low contrast due to scatter shading effect, which is more obvious in the line profile of Fig 9. The overestimated scatter signal in the SVSC method results in severe streaking artifacts (Fig 8C), which is anticipated from the artifacts in the corresponding projection image (Fig 7B). These artifacts are effectively suppressed by the MVSC method using five adjacent views (MVSC, Fig 8D and 8H). The line profiles along the middle of the transverse slice of Fig 8 are shown in Fig 9. MVSC (red line) improved the contrast compared to that without SC (black line). However, SVSC (green line) suffers not only high noise due to the streaking artifacts, but also overcorrected CT numbers, especially for the center air part (30~50% less than -1000 HU). MVSC method significantly improved the contrast without introducing notable noise.

### 3.2 Scatter estimation and correction for the pelvis phantom reconstruction

#### 3.2.1 Boundary effect

**Fig 10:**
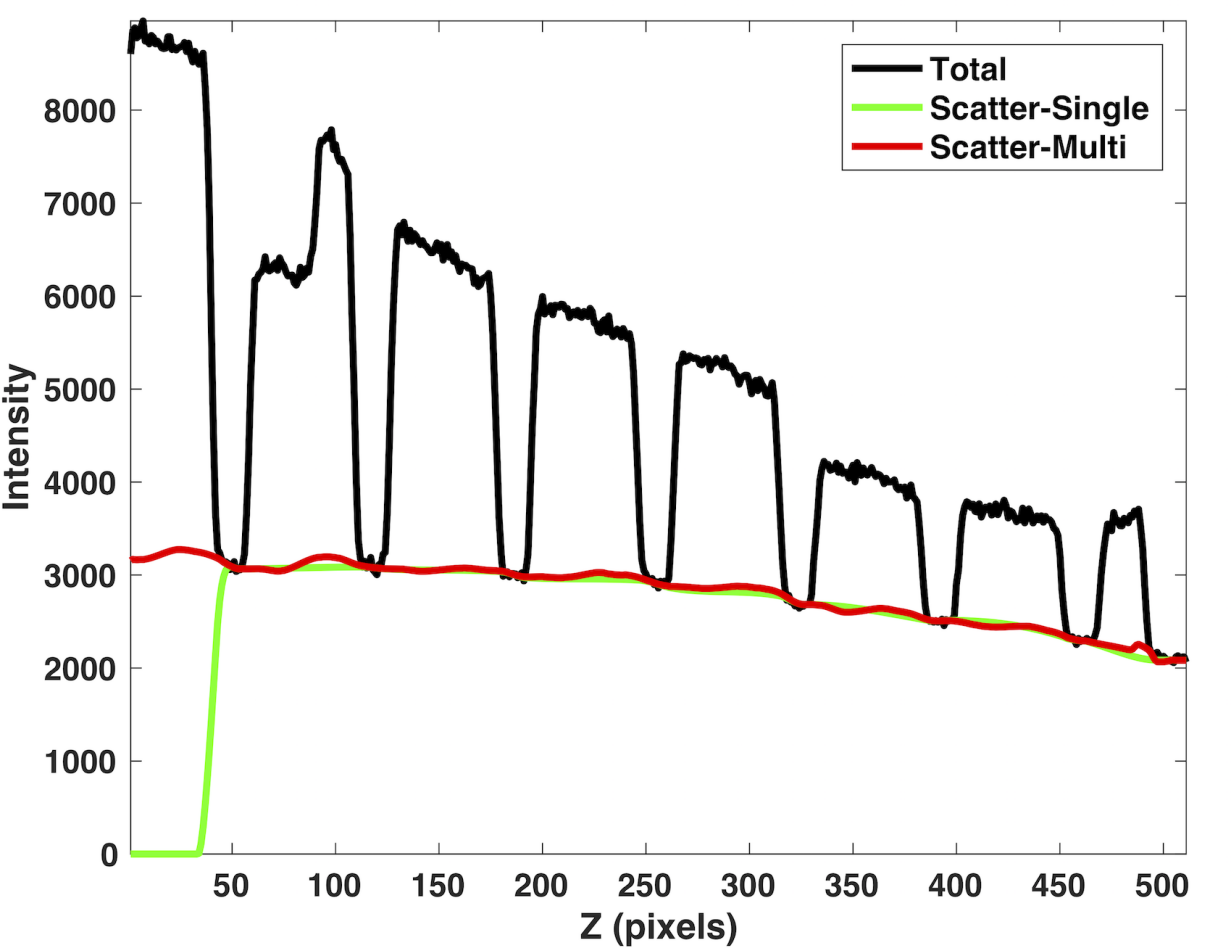
The comparison of the scatter signal profiles estimated from SVSC (green line) and MVSC (red line) when the left marginal region is lack of scatter samples for the anthropomorphic pelvis phantom (9.6 mm pitch blocker). The observed total signal is in black.

Fig 10 shows the estimated scatter profiles in one row of a projection image using the SVSC method (green line) and the MVSC method using 5 adjacent views (red line) along with the observed total signal (black line) for the pelvis phantom. The scatter signal is underestimated for the SVSC method in the leftmost unblocked region due to the lack of scatter signal samples to the left. The similar erroneous scatter estimation is also observed for other rows in the same projection image as well as other projection images in this region.

**Fig 11:**
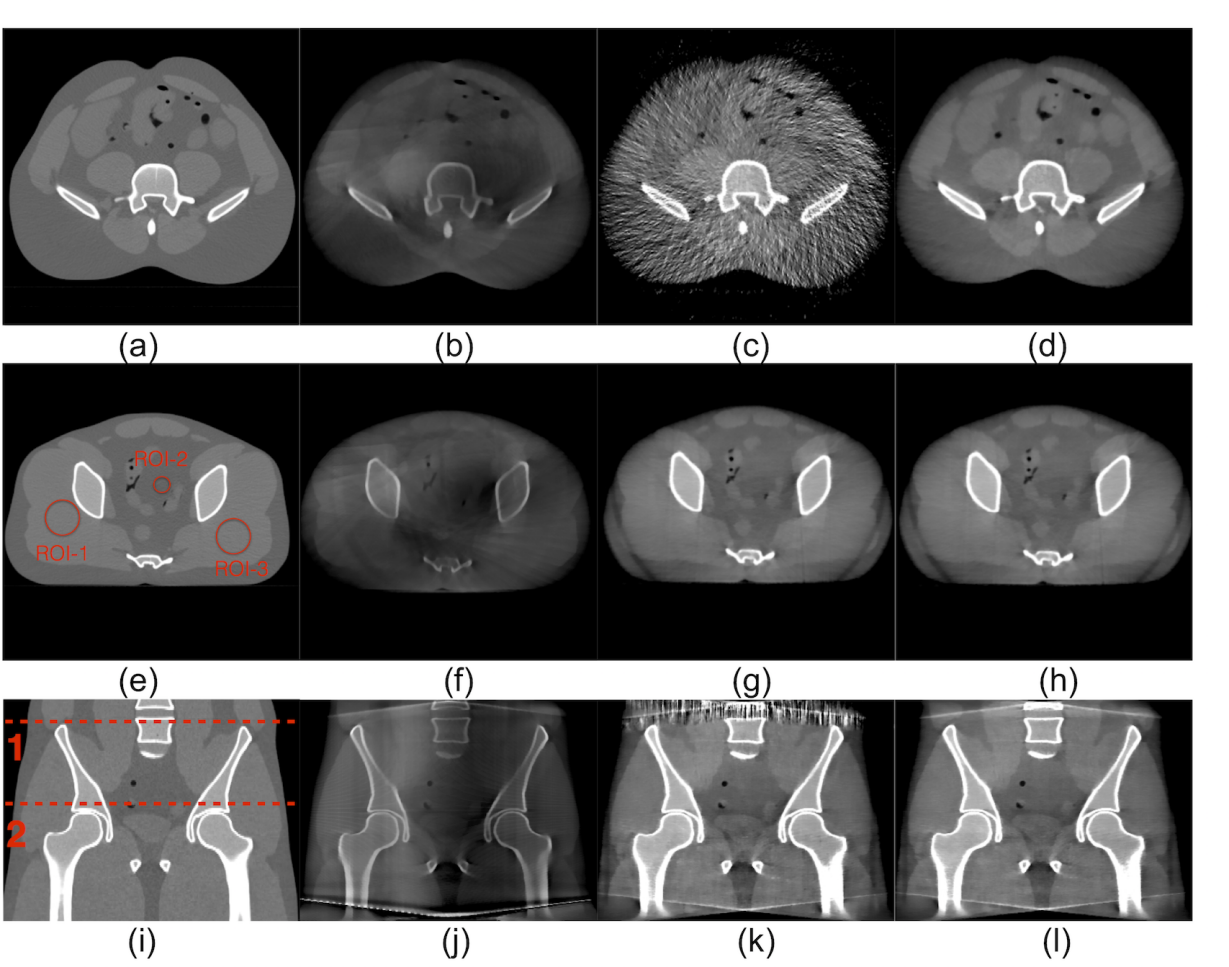
Edge effect: Two transverse slices (in the top and middle row) and one coronal slice (in the bottom row) of the anthropomorphic pelvis phantom. First column (a, e, i): MDCT; second column (b, f, j): CBCT without SC; third column (c, g, k): CBCT with SVSC; fourth column (d, f, l): CBCT with MVSC. The locations of the transverse slices are labeled in (i) (1 for the top row and 2 for the middle row). Regions of interest (ROIs) labeled in (e) were used for measuring the reconstruction accuracy quantitatively. (Display window [−600, 800] HU).

In Fig 11, two transverse slices (the top and middle rows) and one coronal slice (the bottom row) are shown for MDCT (Fig 11A, 11E and 11I), CBCT without SC (Fig 11B, 11F and 11J), CBCT with SVSC (Fig 11C, 11G and 11K), and CBCT with MVSC (5 views) (Fig 11D, 11H and 11L) from left to right (display window [–600 800] HU). The locations of the transverse slices are labeled as the red dashed lines 1 and 2 on the coronal slice of MDCT (Fig 11I). The scatter shading artifacts, especially at the central soft tissues, can be seen in CBCT without SC (Fig 11B, 11F and 11J) and lead to greatly reduced image contrast. The erroneous scatter estimation at the boundary can introduce severe streaking artifacts for the slices in these regions in the reconstructed image with SVSC as shown in Fig 11C. The proposed MVSC method effectively eliminates these artifacts and largely recovers the image contrast lowered by scatter contamination in CBCT (Fig 11D). For both SVSC and MVSC, the middle transverse slices (Fig 11G and 11H) show improved contrast compared to CBCT without SC. In addition, the MVSC image (Fig 11L) seems to be less noisy than the SVSC (Fig 11K) in the coronal view. Note that achieving a larger FOV of MVSC at the same radiation dose of SVSC is clinically significant.

The mean and standard deviation (STD) of CT numbers in the ROIs delineated in Fig 11E are shown in Table 1 as well as the RMSE values between different CBCT reconstructions (without SC (WOSC), SVSC, and MVSC) and MDCT. Both SVSC and MVSC greatly reduce the bias (the deviation of the mean from that of MDCT) in all ROIs (with only a little STD increase in ROI-1 and ROI-3). Consequently, the RMSE values are largely reduced from 379 (WOSC) to 29 (SVSC) and 29 (MVSC) for ROI-1, from 304 (WOSC) to 32 (SVSC) and 31(MVSC) for ROI-2, from 305 (WOSC) to 35 (SVSC) and 34 (MVSC) for ROI-3. These results demonstrate that MVSC not only eliminates the artifacts caused by the lack of scatter signal samples in the boundary region, but also maintains the superior scatter correction performance of the original SVSC method for the other regions.

**Table 1:** Comparison of the CT number (HU) of three ROIs of the Pelvis phantom in Fig 11.

		ROI-1			ROI-2			ROI-3	
	Mean	STD	RMSE	Mean	STD	RMSE	Mean	STD	RMSE
MDCT	72	15		-48	18		70	19	
WOSC	-308	23	379	-348	47	304	-234	19	305
SVSC	71	26	29	-46	30	32	67	35	35
MVSC	71	26	29	-44	29	31	71	34	34

#### 3.2.2 Robustness

**Fig 12:**
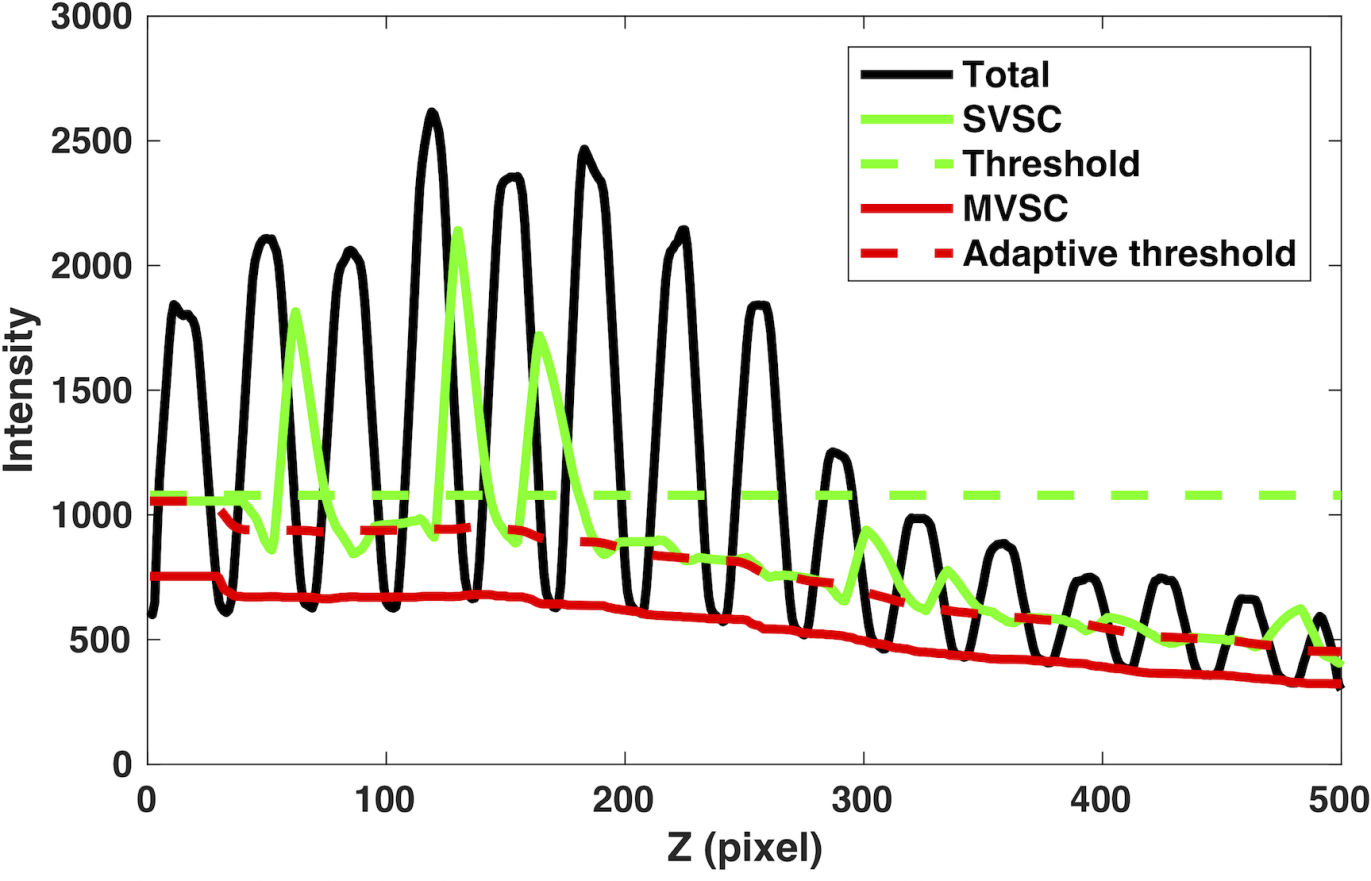
The comparison of scatter signal profiles estimated from SVSC (solid green lines) and MVSC (solid red lines) when the blocker edge detection error occurs for the anthropomorphic pelvis phantom (3.2 mm pitch blocker). The red dashed line and green dashed line are the derived adaptive threshold and the fixed threshold (mean of the profile) for blocker detection, respectively. The observed total signal is in black.

Fig 12 shows one estimated scatter profile using the SVSC method (green solid line) and the MVSC (5 adjacent views) method (red solid line) along with the observed total signal (black line). The erroneous blocker edge detection in one projection view caused that the signal in the unblocked regions was counted as the scatter signal. It thus introduced large errors in the estimated scatter signal by the SVSC method (green solid line in Fig 12). In contrast, if the neighboring views are taking into account, the MVSC method can utilize signal profiles from these views to robustly estimate the scatter signal as shown as red dashed line in Fig 12 without the need of blocker detection. It is important to note that the MVSC method can utilize the derived scatter profile to generate adaptive threshold (red dashed line) and to achieve more accurate edge detection. Thus, the primary signal in unblocked regions can be recovered more reliably.

**Fig 13:**
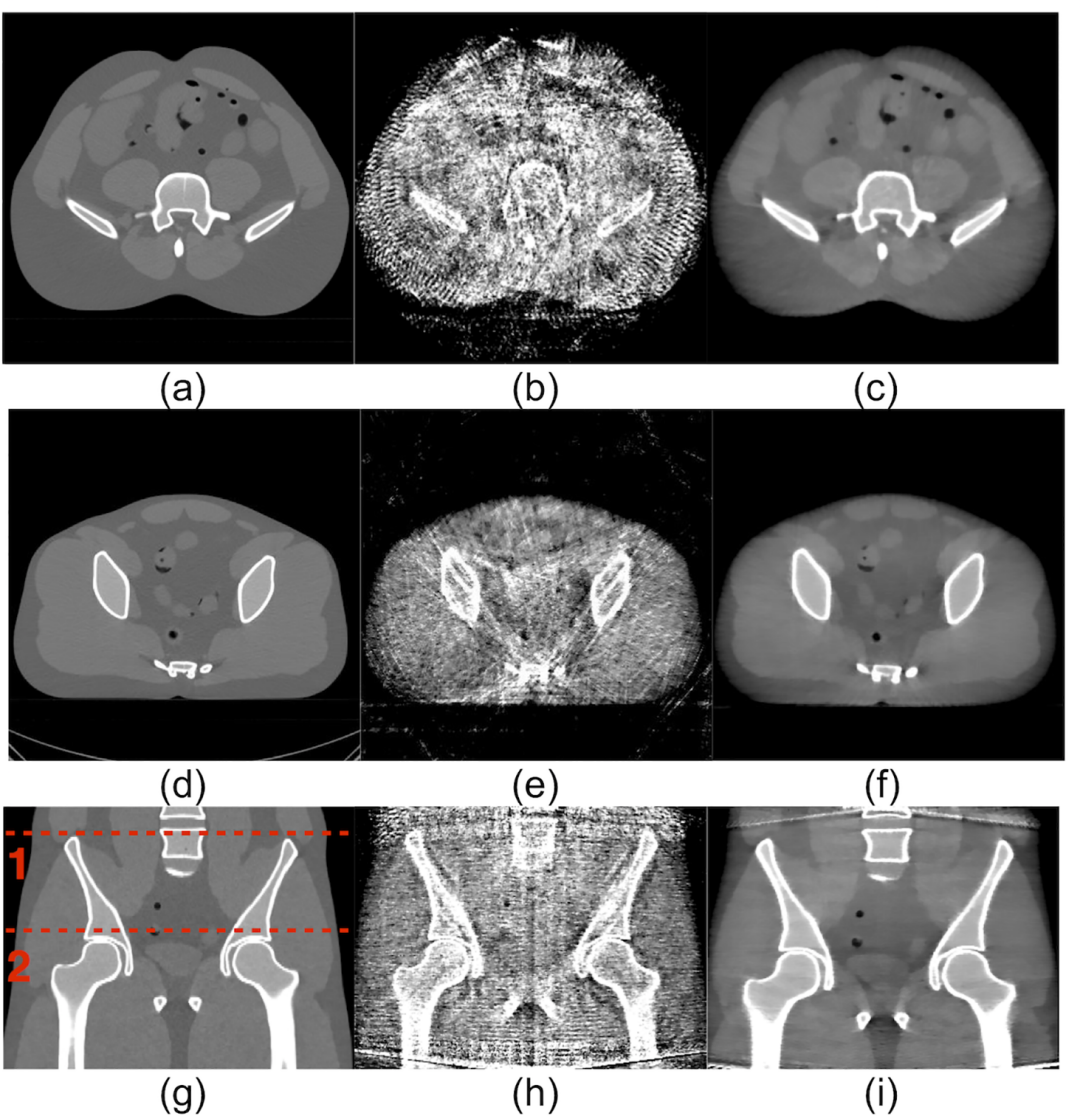
Robustness: Two transverse slices (in the top and middle row) and one coronal slice (in the bottom row) of the anthropomorphic pelvis phantom. First column (a, d, g): MDCT; second column (b, e, h): CBCT with SVSC; third column (c, f, i): CBCT with MVSC of five adjacent views. The locations of the transverse slices are labeled in (g) (1 for the top row and 2 for the middle row). (Display widow [−600, 800] HU).

Two transverse slices (the top and middle rows) and one coronal slice (the bottom row) are shown in Fig 13 for MDCT, SVSC, MVSC (using five adjacent views) from left to right (display window [–600 800] HU). The locations of the transverse slices are labeled as the red dashed lines 1 and 2 on the coronal slice of MDCT. Both primary signal and scatter signal estimation errors in SVSC resulted in severe streak artifacts and underappreciated contrast (Fig 13B, 13E and 13H). The MVSC (Fig 13C, 13F and 13I) effectively corrected such errors and greatly improved the image quality with less streaking artifacts and enhanced contrast. It is noted that this set of data using the small pitch value (large spatial sampling rate) so that the boundary effect has been avoided.

## 4. Discussion

In this study, we investigated the scatter estimation errors caused by single-view SC (SVSC) used in the moving-blocker SC method for the regions with fast scatter signal changes or lack of scatter samples and the blocker detection errors. To make the moving blocker SC method suitable for these regions and robust to the blocker detection errors, we proposed a multi-view SC (MVSC) method using several neighboring views.

The scatter estimation and corresponding reconstruction results show that the MVSC method can eliminate the scatter estimation errors in the regions with fast scatter signal changes (Fig 6) or lack of scatter samples (Fig 10) and maintain the superior scatter correction performance for other regions (Table 1). In the regions with fast scatter signal changes or missing scatter samples at the margin, the scatter signal is hard to be recovered accurately with limited sampling points by using just one projection view in SVSC. The MVSC method uses the adjacent projection views, which can provide more information of the scatter signal in different locations along the blocker moving direction because of the blocker movement during the gantry rotation. The scatter signal is recovered more accurately with the MVSC method and result in a reconstructed image with reduced artifacts and more accurate quantitative results (CT numbers).

Furthermore, the accurate blocker detection is required in SVSC not only for accurate scatter signal estimation in the blocked regions, but also for the accurate primary signal estimation in the unblocked regions. However, the blocker detection error may occur in certain views, especially for large objects where the projection image contrast is low. A robust blocker edge detection and scatter estimation method is in need to improve the clinical translation of the moving-blocker SC method. The proposed MVSC method can use multi-view information to achieve accurate scatter profile estimation without edge detection at first. Consequently, the estimated scatter profile can be used for an adaptive thresholding for more accurate and robust edge detection for primary signal estimation, thus leading to much improved reconstruction image quality. In order to make the proposed method work as shown in Fig 5, the detected scatter signal from several adjacent projection views shall not have much difference and must overlap with each other, which is often the case for a slow moving blocker with a small pitch.

The number of adjacent projection views that can be used in the MVSC methods was also investigated in this study. For the data acquired in this study, if the angle spanned by the adjacent views is less than 3^o^ (corresponding to maximum 5 adjacent views in a 0.5 ^o^ gantry angular sampling), negligible scatter difference is observed among the adjacent projections and leads to superior MVSC performance as shown in Fig 11, Fig 13 and Table 1. As more adjacent views were used, the MVSC performance deteriorates due to the increased discrepancy of the scatter signals in these views. The optimal blocker pitch and speed were investigated in another study for SVSC [36] and will be further studied for MVSC in future.

**Fig 14:**
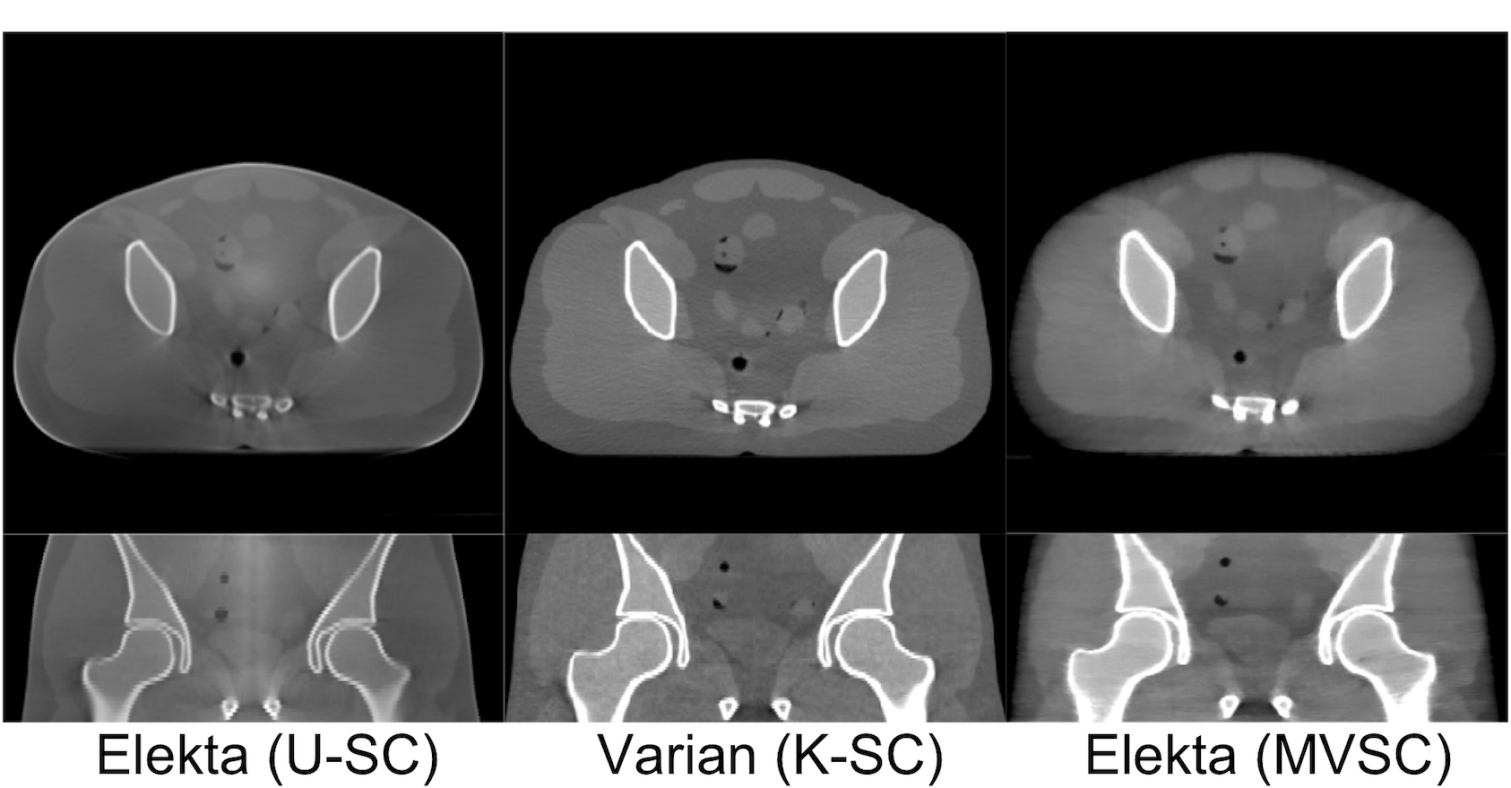
Comparison between the proposed MVSC method and the clinical SC methods (U-SC: Uniform scatter correction; K-SC: Kernel-based scatter correction).

**Fig 15:**
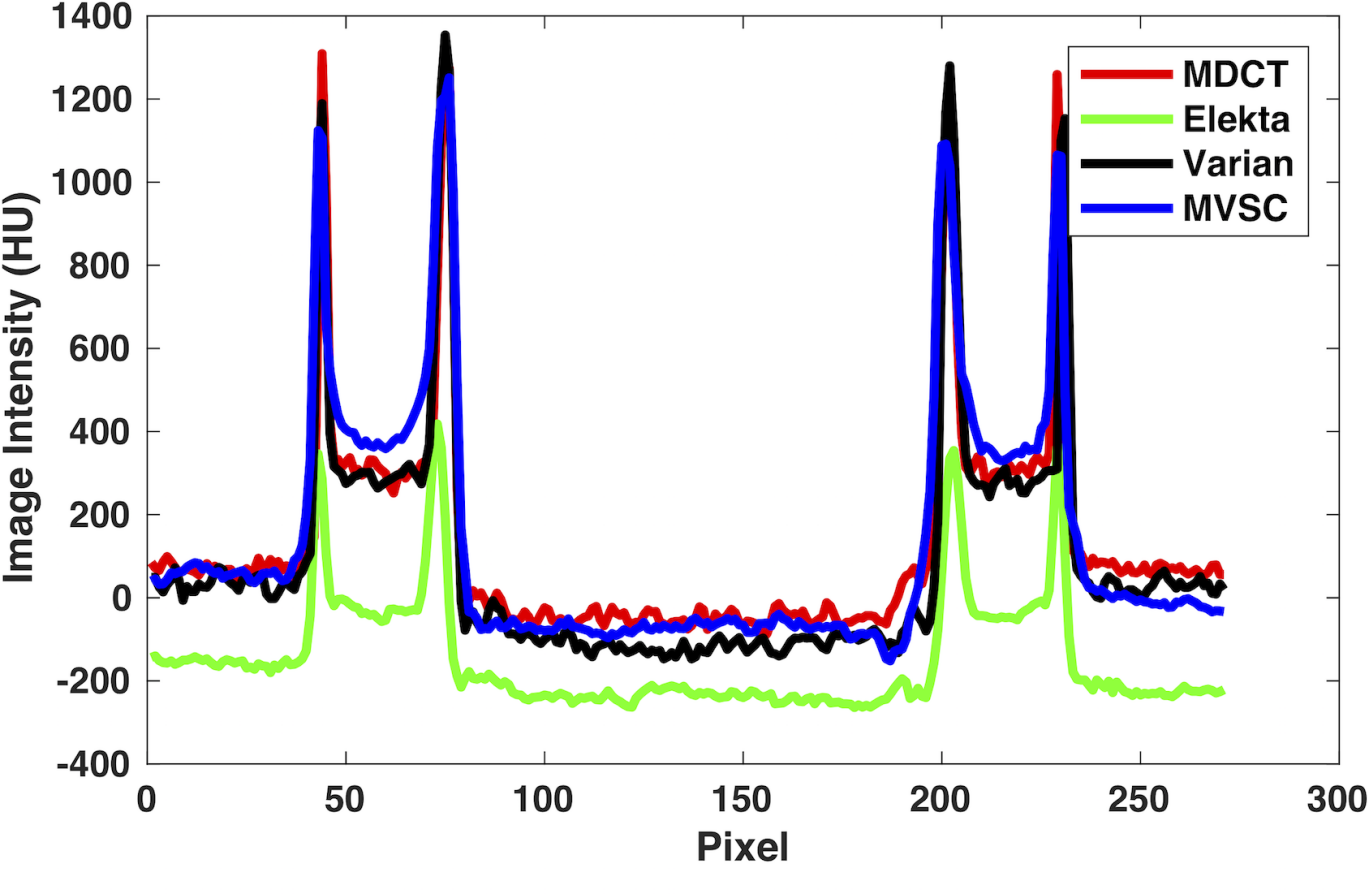
Comparison of the horizontal line profile (red line in Fig 14) for Elekta with U-SC, Varian with K-SC and MVSC. The MDCT profile serves as the ground truth.

In this study, we mainly focus on improving the conventional moving blocker method and compare between SVSC and MVSC. Although it would be an interesting topic to investigate the performance of different SC methods, such as analytic modeling and Monte Carlo simulation, it is out of the scope of this work. To shed light on how the moving blocker based method compared to the clinical routine, kernel-based scatter correction (K-SC) used on Varian TrueBeam OBI (125kVp, 60mA/20ms) and uniform scatter correction (U-SC) used on Elekta XVI (120 kVp, 40 mA/40 ms), we show a set of images in Fig 14. (At this point, the moving blocker can be only mounted on the Elekta system). All these images have less shading artifacts than that without SC (Fig 11J). The proposed MVSC method and the K-SC method outperform the U-SC method. The images from Varian using K-SC seem to have better contrast than MVSC on Elekta, partially due to the use of anti-scatter grid and additional image processing employed by Varian OBI, such as analytical beam hardening correction, additional noise suppression technique and ring artifacts correction [37–39]. More importantly, heavier spatial regularization is deployed in the MVSC method to alleviate the missing data problem caused by ~50% of the incident X-ray blocked by the moving blocker in this example. The MVSC method is indeed beneficial to the patient since the radiation dose is greatly reduced. With additional image processing and increased mAs to compensate the loss of the detected photon flux due to the blocker (without increasing the radiation dose), it is envisioned that the MVSC method can produce comparable images to Varian with K-SC, if not better. Nevertheless, the shading artifact is effectively removed in both Varian with K-SC and the proposed MVSC method as shown in the line profiles of Fig 15.

Finally, the MVSC method is performed in the projection domain. The increased computational time due to use of multiple views instead of a single view is negligible compared to the reconstruction time. Thus, it is a practical SC method in terms of the computational complexity.

## 5. Conclusions

The results from the phantom studies show that the proposed MVSC method allows the moving blocker SC method to estimate the scatter signal more accurately in the regions with fast scatter signal changes or lack of scatter samples in the current view and to correct blocker detection errors caused by the two-point method. This development will expand the utility of moving blocker-based SC for the target with sharp intensity changes in the projection images and make it more robust to blocker detection errors. The further investigation is planned to test the MVSC method on the phantoms, such as the chest and the head, where the bone/lung and body/air interfaces may manifest its benefit.
